# Distance matters: a population based study examining access to maternity services for rural women

**DOI:** 10.1186/1472-6963-11-147

**Published:** 2011-06-10

**Authors:** Stefan Grzybowski, Kathrin Stoll, Jude Kornelsen

**Affiliations:** 1 Centre for Rural Health Research, Department of Family Practice, University of British Columbia, Vancouver, Canada

## Abstract

**Background:**

In the past fifteen years there has been a wave of closures of small maternity services in Canada and other developed nations which results in the need for rural parturient women to travel to access care. The purpose of our study is to systematically document newborn and maternal outcomes as they relate to distance to travel to access the nearest maternity services with Cesarean section capabililty.

**Methods:**

Study population is all women carrying a singleton pregnancy beyond 20 weeks and delivering between April 1, 2000 and March 31, 2004 and residing outside of the core urban areas of British Columbia. Maternal and newborn data was linked to specific geographic catchments by the B.C. Perinatal Health Program. Catchments were stratified by distance to nearest maternity service with Cesarean section capabililty if greater than 1 hour travel time or level of local service. Hierarchical logistic regression was used to test predictors of adverse newborn and maternal outcomes.

**Results:**

49,402 cases of women and newborns resident in rural catchments were included. Adjusted odds ratios for perinatal mortality for newborns from catchments greater than 4 hours from services was 3.17 (95% CI 1.45-6.95). Newborns from catchments 2 to 4 hours, and 1 to 2 hours from services generated rates of 179 and 100 NICU 3 days per thousand births respectively compared to 42 days for newborns from catchments served by specialists.

**Conclusions:**

Distance matters: rural parturient women who have to travel to access maternity services have increased rates of adverse perinatal outcomes.

## Background

In the past fifteen years there has been a wave of closures of small rural maternity services [1-3]. This is coincident with the regionalization of health services world-wide and the concomitant challenges in recruiting and retaining rural providers [4-6]. The closure of such services results in the need for rural parturient women to travel to access care, [7,8] this being most challenging for socioeconomically vulnerable women and families who have the most difficulty in mobilizing the financial and support resources needed to travel to access services in a referral centre [9]. Lack of local access to care in rural environments has previously been associated with negative perinatal outcomes [10,11]. Larimore modelled the potential increase in perinatal mortality associated with the loss of maternity care providers in rural Florida and found a 9% increase was associated with the loss of local specialist obstetrical care [10]. A larger population based study has shown a slightly increased level of risk (RR 1.4) for term newborns born to women who live in communities served by a small hospital (< 100 per year) compared to those in communities served by a large hospital (> 2000 births) [12]. While this slight but important difference supports the supposition that it may be safer to live in or near a facility providing a larger volume of maternity services it does not address the potential adverse outcomes associated with having no access to elective local intrapartum services at all. Two recent publications have found an association between distance to travel to access maternity services and adverse outcomes [13,14].

Recent evidence examining the psychological experience of pregnancy in rural communities has documented a 7 times greater likelihood of increased stress for women who have to travel more than one hour to access maternity services [15]. While the physiological mechanisms are only poorly understood, stress in pregnancy has been associated with a range of complications including preterm labour and birth and spontaneous abortion [16-18].

It is only in recent years that a systematic erosion of long standing basic services in small rural communities has created the opportunity to examine the issue of access in sufficient detail within the Canadian medical context. The purpose of our study is to systematically document newborn and maternal outcomes as they relate to distance to travel to access the nearest referral maternity services.

## Methods

The study setting is British Columbia, the mountainous most western province in Canada with a population of just over 4.5 million scattered over just under 950,000 square kms and a population density of 4.7 per square kilometre [19,20]. Of the approximately 42,000 births recorded annually we have excluded the 27,000 that occur to residents of the urban and suburban areas of the Southwestern segment of the province including Victoria and suburbs, Vancouver and suburbs, and the adjacent Fraser Valley. The health system in British Columbia and across Canada provides universal medical coverage for core health care [21]. Access costs for residents of rural and remote areas are generally the responsibility of the individual though Aboriginal people living on reserve have access to travel subsidies when forced to leave their homes to access medical services [22,23].

British Columbia has categorized Neonatal Intensive Care units (NICU) into either Level 2 (transitioning) or Level 3 (most severely compromised) dependant on the scope of problems (level of prematurity, respiratory status) of the newborns under care in the facility [24]. There are 13 NICU's in the province, 4 of which are located in rural referral centres (Prince George, Kamloops, Kelowna, and Nanaimo).

The study population is taken from the women and newborns residing in communities ranging from small settlements of less than 100 people in remote valleys and coastal enclaves to residents of rural referral centres with populations of about 100,000 such as Prince George, Kelowna and Kamloops. All women carrying a singleton pregnancy beyond 20 weeks and delivering between April 1, 2000 and March 31, 2004 were included in the study. As well as excluding women with twins or multiples, we excluded all recognized congenital anomalies and late terminations from the analysis in order to more clearly focus on the relationship between outcomes and services.

Using data from the BC Perinatal Health Program we defined the location of all rural maternity services in the province and using GIS we created geographic 1 hour travel catchments for each maternity service [25]. In British Columbia rural residents' addresses are generally defined using a 6 digit postal code which translates into a final postal distribution point. While specific street addresses exist at a community level to facilitate emergency access, they are not consistently recorded on birth records. Each postal code has a defined centroid with longitude and latitude coordinates. We defined the distance from each postal code centroid to the nearest maternity service point and grouped all rural postal codes into unique catchments based on proximity to a maternity service level within 60 minutes of surface travel time [25]. We then extended the definition of catchments to women residing 1 to 2, 2 to 4, and greater than 4 hours from the nearest maternity service with Cesarean section capability and defined the postal codes that fell within each catchment.

Maternal and newborn data was linked to specific catchments by the B.C. Perinatal Health Program (BCPHP) using the postal code of maternal residence regardless of the actual location of delivery. Catchments were stratified by distance to nearest maternity service or level of local service as shown in Table 1. When a hospital changed service level during the 4 years of the study we changed the service level designation and linked the data with the appropriate strata.

**Table 1:** Summary of obstetric service levels for Rural British Columbia (2000-2004)

	Obstetric Service Level	Definition of Service Level	# of Catchments	# of Births
**1**	No local services	Greater than 240 minutes (4 Hours) from maternity services	15	506

**2**	No local services	121-240 minutes (2-4 Hours) from maternity services	19	747

**3**	No local services	61-120 minutes (1-2 Hours) from maternity services	23	1,359

**4**	Primary care services with and without Cesarean Section (GP Surgeons)	Intrapartum care provided by Family Physicians and Midwives (No local specialist access)	31	8,031

**5**	Mixed Model	C-section provided by GP surgeon or Specialist	8	5,945

**6**	OB/GYN and General Surgery	C-section provided by Obstetricians or General Surgeons	19	32,814

	**Total**		115	**49,402**

To test for significant associations among obstetric service levels and maternal characteristics, risk factors, and perinatal outcomes, a one way Anova was performed for continuous variables and the chi square test for categorical variables. P values of < 0.05 were deemed significant. Findings are displayed in Table 2.

**Table 2:** Maternal characteristics and ecological determinants by obstetric service level (N = 49,402)

Service Level	240+ minutes from services	120-240 minutes from services	60-120 minutes from services	Primary care	Mixed model	Specialist C/S Services	P-Value
**Maternal characteristics**							

Average maternal age	27.23	27.25	28.65	27.81	27.67	28.65	< 0.001

% Nulliparous women	36.8	36.7	38.6	38.4	40.9	42.6	< 0.001

Antepartum hemorrhage at or > 20 wks (%)	0.8	0.8	1.9	1.1	1.2	1.0	0.037

Pregnancy induced hypertension (%)	3.8	4.7	3.8	4.4	4.5	4.5	0.732

Preexisting and/or gestational diabetes (%)	2.4	3.6	3.2	3.0	2.5	4.1	< 0.001

Smoking during pregnancy (%)	20.6	16.5	16.5	20.3	18.0	17.9	< 0.001

Alcohol use during pregnancy (%)	6.5	3.2	1.5	1.3	1.7	1.6	< 0.001

**Ecological determinants**							

Catchment level social vulnerability-1 to +1 [30]	0.33	0.30	0.10	0.23	- 0.002	0.12	< 0.001

Catchment proportion of Aboriginal people	0.42	0.23	0.30	0.13	0.08	0.05	< 0.001

We performed hierarchical logistic regression analyses to examine the effect of obstetric service level on maternal and newborn outcomes. The newborn outcomes of interest were perinatal mortality (including stillbirths, and early neonatal mortality), prematurity (gestational age < 37 weeks), and admissions to the neonatal intensive care unit. The maternal outcomes we examined were induction of labour, primary Cesarean section, and unplanned out of hospital deliveries. We controlled for two sets of variables: maternal characteristics and risk factors (entered in step 1 of the logistic regression model), and ecological determinants of outcomes, i.e. catchment level social vulnerability and proportion of Aboriginal people residing within the catchment (entered in step 2). The primary maternal characteristics that were entered into all six models were maternal age over 35 or under 16 and parity. We also controlled for the following pre-existing or pregnancy induced medical conditions when assessing predictors of adverse newborn outcomes: pre-existing and/or gestational diabetes mellitus (I and II) and antepartum hemorrhage at equal or greater than 20 weeks gestation. Because we could detect no significant differences in the prevalence of pregnancy induced hypertension across service levels, this medical condition was not included in the regression models. For the logistic regression model testing predictors of perinatal mortality, a history of stillbirths and a previous neonatal death were added as control variables. For the logistic regression model testing predictors of prematurity, history of premature birth was added as a control variable. Maternity care service level was dummy coded into 5 levels plus one reference category (level 6) which was the highest level of service available in communities in our study. Social vulnerability scores ranged from -1 to +1 with scores closer to +1 indicating increased vulnerability [26]. The proportion of Aboriginals variable had a theoretical range from 0-1. Analysis was done using SPSS software (Version 18). Ethical approval for the study was granted by the University of British Columbia ethics board.

## Results

From April 1, 2000 to March 31, 2004, BCPHP provided us with maternal and newborn data for 52, 139 deliveries. After exclusions there remained 49,402 cases of women and newborns resident in the rural population catchments. Table 1 presents the distribution of women by service level in their home communities. Over 5% of parturient women resided in catchments with no local access to intrapartum services within one hour travel time.

Table 2 compares selected characteristics of women across service strata including age and parity and ecological profiles such as socio-economic status and ethnicity.

Table 3 compares newborn outcomes across service strata. After adjustment for potential confounding factors Table 4 demonstrates an odds ratio of 3.17 (95% CI 1.45- 6.95) for perinatal mortality for births from level 1 communities (> than 4 hours from intrapartum services).

**Table 3:** Neonatal outcomes compared across obstetric service levels

Service Level	240+ minutes from services	120-240 minutes from services	60-120 minutes from services	Primary care	Mixed model	Specialist C/S Services	P-Value
% of deliveries at facilities with NICU beds	39.9	12.0	46.9	16.1	2.8	51.6	< 0.001

Perinatal mortality per 1000 births	18	5	6	9	8	6	0.001

Birth weight < 2500 gr per 1000 births	36	24	43	36	34	38	0.253

Gestational Age < 37 weeks per 1000 births	86	71	86	63	65	71	0.004

NICU 2 admissions per 1000 births *	27	11	51	26	8	33	< 0.001

NICU 3 admissions per 1000 births*	4	5	8	3	2	3	0.004

NICU 2 days per 1000 births*	140	141	480	189	80	229	< 0.001

NICU 3 days per 1000 births*	8	179	100	24	34	42	0.007

*NICU 2 and 3 data only available from 2001-2004

**Table 4:** Hierarchical logistic regression results for newborn outcomes

	Perinatal Mortality N = 49,402		Prematurity N = 49,402		NICU 2 Admissions N = 36,805	
	**OR**	**95% CI**	**OR**	**95% CI**	**OR**	**95% CI**

**Step 1: Maternal Risk Factors**						

Maternal Age < 16 or > 35	1.62	(1.23, 2.14) **	1.25	(1.14, 1.38) ***	1.22	(1.03, 1.45)*

Nulliparity	1.38	(1.09, 1.72) **	1.33	(1.24, 1.45) ***	1.40	(1.23, 1.58) ***

Previous stillbirth	2.68	(1.30, 5.54) **	NA	NA	NA	NA

Previous neonatal death	2.25	(0.81, 6.18)	NA	NA	NA	NA

Previous premature birth	NA	NA	4.49	(4.00, 5.03) ***	NA	NA

Antepartum hemorrhage > 20 wks	8.29	(5.51, 12.44) ***	7.50	(6.23, 9.03) ***	5.97	(4.41, 8.10) ***

Diabetes (Pre-existing and/or gestational)	0.79	(0.42, 1.49)	1.87	(1.62, 2.17) ***	3.17	(2.57, 3.91) ***

**Step 2: Ecological risk factors**						

Social vulnerability	1.86	(1.24, 2.78) **	1.12	(0.99, 1.27)	8.79	(6.91, 11.17) ***

Proportion Aboriginal	0.73	(0.24, 2.24)	1.18	(0.79, 1.76)	0.19	(0.09, 0.39) ***

**Step 3: Obstetric Service Level**						

Level 1	3.17	(1.45, 6.95) **	1.11	(0.78, 1.59)	1.07	(0.54, 2.12)

Level 2	0.92	(0.33, 2.53)	0.94	(0.70, 1.27)	0.31	(0.14, 0.65) ***

Level 3	1.04	(0.48, 2.22)	1.12	(0.89, 1.41)	2.20	(1.59, 3.05) ***

Level 4	1.44	(1.08, 1.93)*	0.85	(0.77, 0.95) **	0.70	(0.58, 0.85) ***

Level 5	1.46	(1.05, 2.03)*	0.89	(0.79, 0.99)*	0.35	(0.25, 0.50) ***

* P-value < .05 ** P-value < .01 *** P-value < .001

NA - the variable was not included in the model as the clinical association was not felt to be strong enough to justify inclusion.

**Figure 1:**
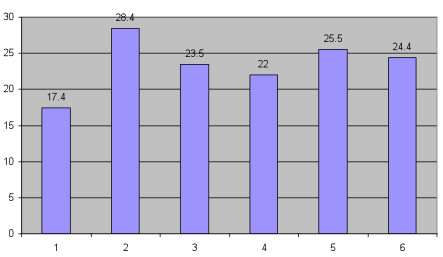
**Induction rates compared across obstetric service levels**.

**Figure 2:**
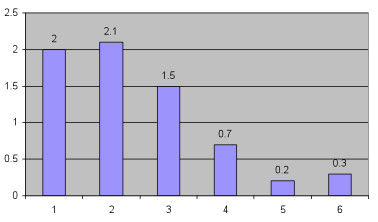
**Rates of induction for logistical reasons compared across obstetric service levels**.

Table 5 summarizes the main outcomes including intervention rates for mothers across service strata. Figure 1 demonstrates the induction rates across service levels and shows that rates are the highest for women travelling 2 to 4 hours to access services. Figure 2 looks specifically at induction for logistical reasons and shows that this is also highest in women travelling 2 to 4 hours to access services. Table 6 shows that the odds ratio for having an unplanned out of hospital birth is 6.41 (95% CI 3.69, 11.28) for women 1 to 2 hours away from services.

**Table 5:** Maternal intervention rates and outcomes across obstetric service levels

Service Level	240+ minutes from services	120-240 minutes from services	60-120 minutes from services	Primary care	Mixed model	Specialist C/S Services	P-Value
% of women who delivered at level 6 hospitals	75.5	67.2	86.5	30.6	18.8	95.8	< 0.001

% Epidural	19.8	17.3	16.6	13.6	17.8	23.7	< 0.001

% Induction (excl. women with planned CS)	17.4	28.4	23.5	22.0	25.5	24.4	< 0.001

% Logistics as reason for induction	2	2.1	1.5	0.7	0.2	0.3	< 0.001

% Episiotomy (vag deliveries only)	7.3	11.2	9.9	11.1	13.4	12.8	< 0.001

% CS (all types)	19	20.9	23	23.7	24.8	26.2	< 0.001

% Planned CS (primary and repeat)	5.5	8.0	8.2	9.3	9.6	9.8	0.006

% Assisted vag. delivery	6.1	6.2	7.5	8.0	9.0	7.9	0.014

% Unplanned out of hospital birth	1.4	0.3	2.3	0.3	0.3	0.2	< 0.001

Average PP length of stay (hours)	51.88	55.03	53.50	53.86	59.05	57.58	< 0.001

Average PP length of stay (hours) CS only	92.30	84.48	83.31	78.92	86.65	84.06	< 0.001

Average PP length of stay (hours) Vag delivery only	41.83	47.22	44.43	45.88	49.71	48.04	< 0.001

**Table 6:** Hierarchical logistic regression results for maternal outcomes

	Primary Cesarean section^1^ N = 43,122		Induction of Labour^2^ N = 44,677		Out of Hospital Deliveries^3^ N = 49,402	
	**OR**	**95% CI**	**OR**	**95% CI**	**OR**	**95% CI**

**Step 1: Maternal Risk Factors**						

Maternal Age < 16 or > 35	1.72	(1.60, 1.85) ***	1.08	(1.01, 1.15) *	0.94	(0.61, 1.45)

Nulliparity	5.50	(5.18, 5.83) ***	1.31	(1.26, 1.37) ***	0.30	(0.20, 0.46) ***

**Step 2: Ecological Determinants**						

Social vulnerability	1.09	(0.99, 1.20)	0.84	(0.78, 0.91) ***	0.49	(0.29, 0.83)**

Proportion Aboriginal	1.25	(0.91, 1.71)	0.81	(0.63, 1.06)	4.79	(1.48, 15.53)**

**Step 3: Obstetric Service Level**						

Level 1	0.67	(0.49, 0.90) **	0.74	(0.57, 0.95) *	3.63	(1.40, 9.40) **

Level 2	0.78	(0.61, 0.98) *	1.34	(1.13, 1.59) **	0.92	(0.22, 3.88)

Level 3	0.78	(0.65, 0.94) **	1.01	(0.87, 1.17)	6.41	(3.69, 11.28) ***

Level 4	0.89	(0.83, 0.96) **	0.91	(0.86, 0.97) **	1.40	(0.89, 2.20)

Level 5	0.92	(0.85, 1.00) *	1.05	(0.98, 1.12)	0.99	(0.57, 1.73)

1. Excluding women with a previous CS

2. Excluding women with a planned CS

3. Excluding planned home deliveries with a registered midwife

* P-value < .05

** P-value < .01

*** P-value < .001

Tables 4 and 6 overview the regression analyses for the key outcomes.

## Discussion

This study shows that having to travel to access intrapartum maternity services for rural parturient women is associated with adverse outcomes for newborns and mothers and increased interventions. Even with the relatively small numbers of births we are able to demonstrate statistically significant increases in perinatal mortality for newborns whose mothers reside greater than 4 hours from services and increased rates of NICU 2 admissions and numbers of NICU 2 and 3 bed days generated per thousand births for newborns whose mothers reside 1-2 hours away from services. The costs of neonatal intensive care days are substantial, estimated at $1300 day (public) for an average NICU 2 day and $2500 per day (public) for an average NICU 3 day. These system costs in adverse outcomes and actual dollars spent need to be considered in the planning process and it may well be that within the fiscal constraints of the regional planning process, the value of sustaining small rural hospital maternity services may be greater than previously appreciated. Most importantly the quality of both newborn and maternal outcomes is associated with access to local services. The cost effectiveness of small rural maternity services needs to be compared to other service situations.

While system costs are important, it is also necessary to consider the costs of travel borne by rural women and families who may be forced to leave a rural community at 36 weeks gestational age to await the onset of labour in a referral centre far from home [9]. Costs of travel, accommodation, lost income for both partners and supplemental food costs can be substantial. While First Nation and Inuit Health (FNIH) subsidizes some costs for First Nations families who live on reserve, even this important contribution only goes part way to defray the financial costs of maintaining a family out of the community for several weeks. Perhaps the potential exists for intergovernmental collaborative solutions which may benefit all parties.

The relative distance women have to travel to access services also is associated with different interventions and outcomes. Women who have to travel more than 2 hours are unlikely to remain at home and try and reach the referral hospital when they go into labour. Our results support previous work that has suggested that inductions for logistical reasons are used to try and shorten the stay [27]. Women who live 1 to 2 hours away from services are more likely to remain at home until the onset of labor particularly if they have other children at home and hence are more likely to deliver en route to the hospital. This is also demonstrated in the results and consistent with previous work [28].

Limitations of the study include the necessity of using a partial ecological design in order to include Aboriginal ethnicity and socio-economic status for which data is unavailable at the case level due to privacy constraints. We have adopted an approach similar to that outlined by Tu and Ko in their cogent review of the subject [29].

NICU 2 admission rates are quite variable across the cohorts. As the clinical criteria for newborn admission to a NICU 2 bed are less stringent than admission to a NICU 3 unit admission rates are subject to greater variation related to provider influence. This is demonstrated by the strong association between proportion of a cohort born in a facility with on site access to NICU beds and the number of newborns per thousand admitted to a NICU 2 bed. Importantly this association does not extend to admission to NICU 3 beds for which the criteria are much more stringent [24].

Also of importance is that the results relate to a geographically mountainous and coastal province on the west coast of Canada where seasonal travel can be particularly difficult due to inclement weather.

The implications of this study support improving access to maternity services for women from rural and remote communities. The recently published Rural Birth Index provides a metric for systematically quantifying need for maternity services in rural community populations and defining the appropriate service level for a given population [30]. The Canada Health Act specifies that insured persons must be provided "reasonable access" to insured services. The research underpinning costs and outcomes needs to redefine what is "reasonable" because if it is reasonable then we should act accordingly. How we treat our most vulnerable populations is a measure of the strength of our society. Perhaps the evidence presented in this study contributes to the evolving larger fabric of understanding about the importance and effectiveness of primary care services to population health. If we do not provide local services to rural residents we should take greater responsibility to overcome geographical barriers to access [31].

## Conclusions

Distance matters: rural parturient women who have to travel to access maternity services have increased rates of adverse outcomes and newborns have increased numbers of NICU 2 and 3 care days. Rural parturient women are also subject to increased rates of inductions for logistical reasons and unplanned out of hospital deliveries. Health planners and policy makers need to consider such findings when planning the fate of rural services.

## Competing interests

The authors declare that they have no competing interests.

## Authors' contributions

SG contributed to conceptualizing the study, writing the grant proposal, implementation and data analysis, and was the lead writer of the manuscript. KS carried out the analysis and contributed to the writing of the manuscript. JK contributed to conceptualizing the study, writing the grant proposal, implementation and data analysis, and the writing of the manuscript.

All authors read and approved the manuscript.

## Authors' information

SG, MD, Professor and Co-Director for the Centre for Rural Health Research in the Department of Family Practice. KS is a PhD Candidate and works with the Centre for Rural Health Research in the Department of Family Practice at the University of British Columbia. JK, PhD, is an Assistant Professor and Co-Director of the Centre for Rural Health Research in the Department of Family Practice at the University of British Columbia.

## Pre-publication history

The pre-publication history for this paper can be accessed here:

http://www.biomedcentral.com/1472-6963/11/147/prepub
